# Prevalence of *Mycobacterium avium* in Slaughter Pigs Based on Serological Monitoring Results and Bacteriological Validation

**DOI:** 10.3390/ijerph10094027

**Published:** 2013-08-30

**Authors:** Anne Hiller, Derk Oorburg, Henk J. Wisselink, Conny B. van Solt-Smits, Bert Urlings, Günter Klein, Gereon Schulze Althoff, Lourens Heres

**Affiliations:** 1 University of Veterinary Medicine, Bischofsholer Damm 15, Hannover 30173, Germany; E-Mail: guenter.klein@tiho-hannover.de; 2 Animal Science Group of Wageningen UR, P.O. Box 338, Wageningen 6700 AH, The Netherlands; E-Mail: bert.urlings@wur.nl; 3 VION Food Group, P.O. Box 1, Boxtel 5280 AA, The Netherlands; E-Mails: gereon.schhulze.althoff@vionfood.com (G.S.A.); lourens.heres@vionfood.com (L.H.); 4 Central Veterinary Institute of Wageningen UR, P.O. Box 65, Lelystad 8200 AB, The Netherlands; E-Mails: henk.wisselink@wur.nl (H.J.W.); conny.vansolt@wur.nl (C.B.S.-S.)

**Keywords:** *Mycobacterium avium*, pig, serology, ELISA, supply chain meat inspection

## Abstract

*Mycobacterium avium* (MA) is a potential food safety hazard in pigs. Blood samples of slaughtered pigs in the Netherlands and Germany were tested for the presence of MA antibodies to estimate the serological prevalence in the tested population. In the Dutch and German population 1.0% and 1.7% samples were positive, and 0.5% and 17.4% of the herds were at risk for having a MA infection respectively. The validity of the applied MA-ELISA was evaluated under field conditions. The specificity of the MA-ELISA was high (>98.4%). The average herd sensitivity was 18%. In the affected herds on average 50% of the animals were tested bacteriological positive for MA. It can be concluded that serological screening for the presence of MA antibodies is capable of identifying pig populations that are at risk for a MA infection.

## 1. Introduction

*Mycobacterium avium* subsp. *avium* and *M. avium* subsp. *hominissuis* belong to the *Mycobacterium avium* complex (MAC) and are frequently associated with diseases in animals and humans. MAC is an opportunistic pathogen which leads to disseminated infections with increased morbidity and mortality, particularly in immune-compromised people [1,2]. MAC infections are reported in 30 to 80% of patients with AIDS [3]. MAC also causes chronic pneumonia in elderly people and cervical lymphadenitis in young children between 0 and 5 years of age [4,5].

Pigs have been suggested as a vector for transmission of MA towards humans [6,7,8]. The main route of infection in pigs is via the gastro-intestinal tract [9]. Outbreaks in herds are described after feeding pigs with mycobacteria contaminated peat, compost, bark mulch and sawdust [10,11]. In pigs MA can cause lymphadenitis with granulomatous lesions, especially the submaxillary and mesenteric lymph nodes are affected [9]. European law (EU/854/2004) prescribes the procedures for meat inspection, which includes the incision of the submaxillary lymph nodes and palpation of the mesenteric lymph nodes within the meat inspection at slaughter. One of the aims of this legal requirement is the detection of mycobacterial infections in pigs at slaughter.

However, the incision of the lymph nodes is characterized by relatively high false positive and false negative results for MA [12,13]. In addition, it can cause cross-contamination with other food safety hazards, e.g., salmonella [14,15]. As an alternative for the lesion criterion, the MA-ELISA test was developed to monitor pig herds serological for MA infections [13].

Starting in 2006 blood samples were collected for monitoring MA infections in slaughter pigs in six Dutch and one German slaughterhouse in the framework of a risk-based meat inspection system [16]. In the present paper the serological prevalence in the tested population was estimated and the tested herds were categorized. Risk categorization was based on an aggregate set of results of the MA-ELISA. The validity of the MA-ELISA test was evaluated under field conditions with samples from MA positive and negative herds.

## 2. Experimental Section

### 2.1. Collection of Samples

At every delivery of a consignment of pigs, blood samples were collected randomly from clinically healthy pigs during bleeding. Samples were identified on a herd level. Treated test tubes (10 mL) for serum collection with coagulation inducer were used. Until coagulation, samples were stored at room temperature and then up to analyses at 4 °C. The blood was send to one laboratory that carried out the MA-ELISA. In six Dutch slaughterhouses from January 2007 until June 2010 blood serum samples were taken from 248,325 pigs delivered by 4,830 herds and examined for MA antibodies. In the German slaughterhouse blood serum samples were taken from 57,044 pigs delivered by 1,249 herds from October 2008 until April 2010.

### 2.2. Applied ELISA and Herd Categorization

Development on the basis of a polar lipid fraction from MA, characteristics of the MA-ELISA and its procedures have been previously described by Wisselink *et al.* [13]. The MA-ELISA test results were calculated as percentage positivity (PP). A cut-off value of PP 20 was used. Herds with two or more positive samples out of 36 samples, achieved over at least 12 batches, were considered at risk for MAA.

### 2.3. MA-ELISA Validation

For the validation of the MA-ELISA and the pathological examination of the submaxillary lymph nodes the bacteriological MA examination of the submaxillary and mesenteric lymph nodes was used as the gold standard.

#### 2.3.1. Evaluation of Sensitivity under Field Conditions

To evaluate the MA-ELISA under field conditions, pig herds (*n* = 11) with a high number of positive serum samples and/or granulomatous lymph nodes at meat inspection were pre-selected. To confirm the MA infection status on these farms, fattening pigs (*n* = 22–68 per herd) which were nearly ready for slaughter were subjected to an intradermal tuberculin test into the base of the ear with 0.1 mL Avian Tuberculin PPD (25.000 I.U., ASG, Lelystad, The Netherlands). Evaluation occurred after 36 to 72 h by checking the injection site for signs of induration and erythema. Herds were selected for sample collection when pigs reacted positive in the tuberculin skin test. From these herds blood serum samples and the submaxillary and mesenteric lymph nodes were collected at slaughter. In the laboratory, serum samples were stored at −20 °C until serological analysis and the submaxillary lymph nodes were examined pathologically for caseous malformations and the submaxillary and mesenteric lymph nodes were bacteriologically examined for MA, as described by Wisselink *et al.* [13]. For bacteriological examination Middlebrook 7H10 plates enriched with OADC on Coletsos Osein and on Dubos Tween albumin medium were used. Ziehl-Neelson stain and PCRs were performed to identify colonies.

#### 2.3.2. Evaluation of Specificity under Field Conditions

For evaluation of the specificity of the MA-ELISA under field conditions, pig herds (*n* = 8) with only negative serological samples for MA were selected. From 239 pigs blood samples and the submaxillary and mesenteric lymph nodes were collected at slaughter and examined as described above.

#### 2.3.3. Herd Sensitivity Calculations

A distinction was made between the sensitivity of an individual test, further called carcass sensitivity and the herd sensitivity when the MA-ELISA was used as a herd diagnostic test. The sensitivity as herd diagnostic test was calculated. Herd sensitivity was defined as the probability that a positive herd is diagnosed positive following the evaluation of an aggregated set of serum samples (in this case 36 samples). The herd sensitivity is the probability that the number of positive samples from positive herds is equal to or above a minimum needed number of positive serum samples, here it is calculated for one and two positive samples, where the latest is used in the system.

The probability that from a set of 36 serum samples < 1 or < 2 serum samples are positive can be calculated with the cumulative distribution function of the binomial distribution, Equation (1):

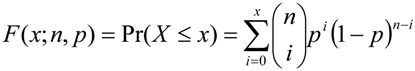
(1)


In that case *n* = 36 and x is respectively 0 and 1. The apparent prevalence in the population (p) is the real prevalence in the population multiplied with the individual carcass sensitivity. The herd sensitivity (Sens_herd_) is the probability that out of the 36 samples more than one respectively two samples are positive:

Sens_herd_ = 1 –Pr (X ≤ x) (2)

The herd sensitivity was calculated for a range of carcass sensitivities at a range of carcass prevalences as observed in the different validation trials.

#### 2.3.4. Herd Specificity Calculations

A distinction was made between the specificity of an individual test, further called carcass specificity and the herd specificity when the MA-ELISA was used as a herd diagnostic test. Herd specificity (Spec_herd_) was calculated in the same way. Herd specificity is one minus the probability that there are two or more false positive test results out of 36 samples calculated with the cumulative distribution function, where probability of a false positive test result is one minus the carcass specificity.

#### 2.3.5. Statistics

To test the statistical significance of differences in proportion of positive samples a Chi square test was done.

## 3. Results and Discussion

### 3.1. Results

#### 3.1.1. Serological MA-Monitoring in the German and Dutch Slaughterhouse(s) and Herd Risk Categorization

**Figure 1:**
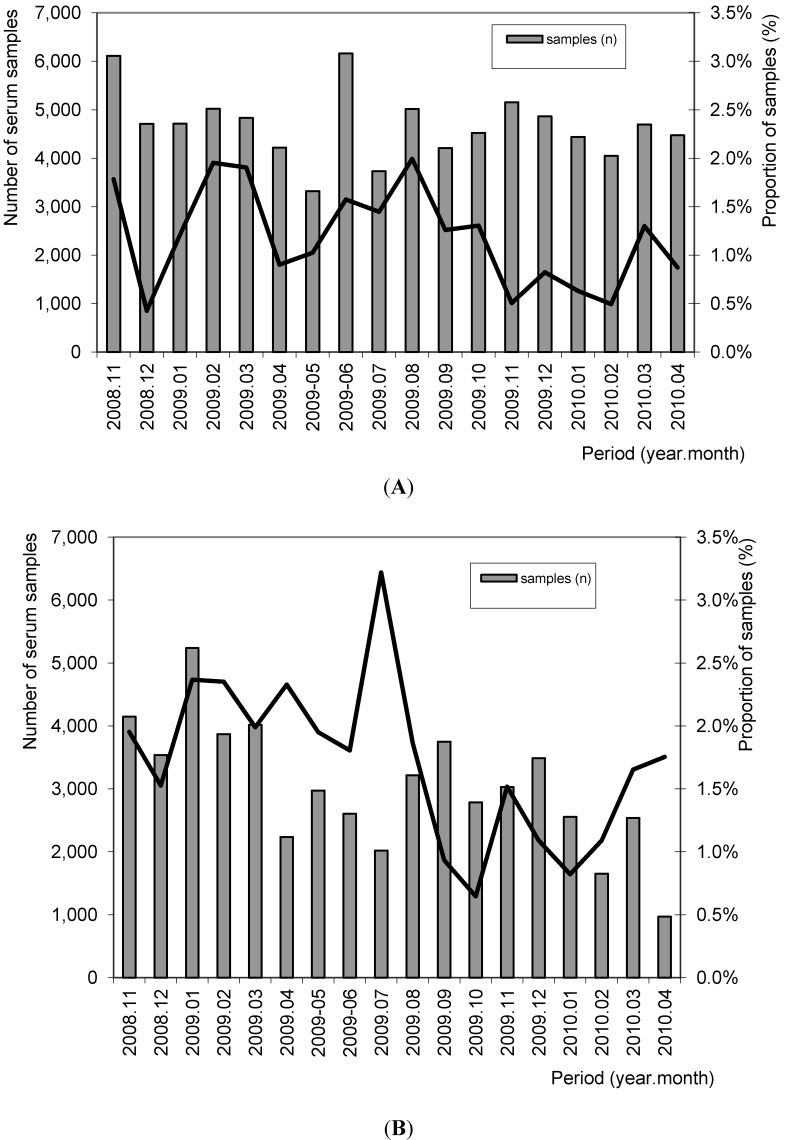
Proportion of MA-ELISA positive serum samples in relation to the number of tested serum samples in The Netherlands (A) and in Germany (B) in the period 2008–2010.

In six Dutch slaughterhouses 248,325 blood serum samples were taken from 4,830 herds and examined for MA antibodies. The results showed that 2,495 (1.0%) serum samples had a positive result in the MA-ELISA (Figure 1). From 4,817 pig herds, at least 36 samples were collected during this period. Twenty five (0.5%) of these herds had two or more positive samples out of 36.

In the German slaughterhouse blood serum samples were taken from 57,044 pigs delivered by 1,249 herds. From these serum samples 984 (1.7%) had a positive result. From 574 herds, at least 36 samples were collected during this period. One hundred of these herds (17.4%) had two or more positive samples out of 36.

The proportion of positive samples in the different months is shown in Figure 1. The proportion of positive samples (1.7% *versus* 1.0%) and herds (17.4% *versus* 0.5%) were significantly higher in the tested German population compared to the tested Dutch pigs (Chi-square; *p* < 0.05).

#### 3.1.2. Evaluation of Sensitivity under Field Conditions

In four of the eleven serologically identified MA farms, pigs were detected with a positive tuberculin skin test. Additional blood and lymph nodes were sampled on pigs from these four tuberculination positive herds (farm A: Netherlands, farm B: Belgium, farm D, E: Germany) and examined for *M. avium* Infections*.*

The results showed that the proportion of the MA-ELISA positive samples at the four farms varied between 3.4% and 11%. The carcass sensitivity (sensitivity of an individual test) based on bacteriological examination of the lymph nodes varied between 2.4% and 16.7% (Table 1). The average proportion of granulomatous lesions detected by pathological examination in the submaxillary lymph nodes was 22.5% (85 of 378). The lowest proportion of lesions was 8% (9 of 117) (farm B) and the maximum was 31% (57 of 184) (farm A). The carcass sensitivity (sensitivity of an individual test) of the pathological examination under laboratory conditions varied between 19.5% and 67% and on average it was 32.6%. Results of bacteriological examination of the submaxillary and mesenteric lymph nodes showed that on average 50% (188 of 375) of the pigs were infected with *M. avium hominisuis* (MAH) (Table 1), the minimum level of infection with MAH was 32% (6 of 19) (farm E) and the maximum level was 66% (38 of 55) (farm D).

**Table 1:** Test characteristics of the applied MA-ELISA validated with animals from four positive farms.

Farm (Country)	Bacteriology ^1^ +ve/n (%)	Pathology^ 2^			Serology (PP > 20)				
		+ve/n (%)	Se%	Sp%	+ve/n (%)	Se% (carcass)	Sp%	PPV%	NPV%
A ^3 ^(NL)	103/184 (56)	57/184 (31)	35.0	74.1	8/184 (4.3)	4.9	96.3	62.5	44.3
B^3^ (B)	41/117 (35)	9/117 (8)	19.5	98.7	4/117 (3.4)	2.4	96.1	25.0	64.6
D ^4^ (GE)	38/55 (66)	16/58 (28)	40.5	94.4	3/55 (5.5)	2.7	88.9	33.3	30.7
E ^4^(GE)	6/19 (32)	3/19 (16)	66.7	92.3	2/19 (11)	16.7	92.3	50.0	70.1
TOTAL (%)	188/375 (50)	85/378 (23)	32.6	87.2	17/375 (4.5)	4.3	95.2	49.4	50.0

^1^ Positive when *M. avium* bacteria were detected by bacteriological examination on submaxillary lymph nodes and/ or mesenteric lymph nodes; ^2^ Positive when granulomatous lesions were seen in the submaxillary lymph nodes during pathological examination in laboratory; ^3^ Partly published by Wisselink *et al.* [13]; ^4^ Partly published by Hiller *et al.* [17] Se = carcass Sensitivity, Sp = carcass Specificity. PPV = Positive Predictive Value, NPV = Negative Predictive Value, +ve = positive, n= number of tested samples B = Belgium, GE = Germany, NL = The Netherlands.

#### 3.1.3. Evaluation of the Carcass Specificity of the MA-ELISA under Field Conditions

From 239 pigs from low MA risk herds the submaxillary and mesenteric lymph nodes were bacteriologically negative for MA. Carcass specificity (specificity of an individual test) of the MA-ELISA at a cut-off of PP 20 was 100% (95% CI: 98.4%–100%). The carcass specificity of the pathological examination under laboratory conditions was 97% (Table 2).

**Table 2:** Results of serological, pathological and bacteriological examinations for *M. avium* infections on farms categorised at “low” risk for a *M. avium* infection.

Pig farm	Number of pigs sampled	Serology	Lnn. mandibulares			Lnn. mesenteriales		
			Pathology ^1^		Bacteriology ^2^	Pathology		Bacteriology
		PP > 20	−ve	+ve	+ve	−ve	+ve	+ve
116	3	0	3	0	0	3	0	0
724	10	0	10	0	0	10	0	0
736	10	0	10	0	0	8	2	0
875	33	0	31	2	0	33	0	0
907	41	0	38	3	0	39	2	0
826	39	0	36	1	0	31	0	0
014	33	0	32	0	0	24	2	0
088	71	0	70	1	0	45	0	0
Total (%)	239	0	230	7 (3.0%)	0	193	6 (3.1%)	0
**Sp**		**100%**		**97%**			**96.9%**	

^1^ Positive when granulomatous lesions were seen in the submaxillary lymph nodes during pathological examination. ^2^ Positive when *M. avium* bacteria were detected by bacteriological examination on submaxillary lymph nodes and/ or mesenteric lymph nodes; −ve = negative, +ve = positive, Sp = Specificity.

#### 3.1.4. Herd Sensitivity Calculations

When the observed range of MA-ELISA carcass sensitivities (2.4%–16.7%) and the observed range for bacteriological carcass prevalence of MA bacteria at herd level (32%–66%) were applied for herd sensitivity calculations, the probability to have at least one positive serological sample varies between 23% and 100% (Table 3). This is the probability that positive herds were recognized with the serological test.

With the average carcass sensitivity of 4.3% and 50% bacteriological positive animals in the affected herds the probability of one positive sample in an affected herd was 54%. The probability to obtain two or more serum samples positive was 3%–97%, depending on the bacteriological prevalence (Table 3). This is the probability for a herd to become categorised as “at risk”. With the average carcass sensitivity of 4.3% and 50% bacteriological positive animals in the affected herds the herd sensitivity was 18%.

#### 3.1.5. Herd Specificity Calculations

Calculations of the apparent herd prevalence (AHP) showed that when the test systematic has a herd sensitivity of 20% and herd specificity of 98.5% the AHP will not be below 1.5%. These were false positives resulting from the 98.5% herd specificity, based on an assumed carcass specificity of 99.5%. In the Dutch population only 0.5% “at risk” farms were detected. Therefor the herd specificity of the test systematic was higher than 98.5%. This also means that the carcass specificity for an individual test was higher than 99.5%.

**Table 3:** Herd sensitivity of the MA-ELISA in a range of observed carcass sensitivities and bacteriological prevalences of *M. avium* at 1 or more and 2 or more positive samples for a positive herd diagnosis.

Within-herd bacteriological prevalence of *M. avium*	MA-ELISA carcass sensitivity				
	2.4	5%	10%	16.7%	20%
	probability ≥ 1 out of 36 positive blood serum samples				
**30%**	23%	42%	67%	84%	89%
**40%**	29%	52%	77%	92%	95%
**50%**	35%	60%	84%	96%	98%
**60%**	41%	67%	89%	98%	99%
**70%**	46%	73%	93%	99%	100%
	**probability ≥ 2 out of 36 positive blood serum samples**				
**30%**	3%	10%	29%	54%	64%
**40%**	5%	16%	42%	70%	79%
**50%**	7%	23%	54%	81%	89%
**60%**	9%	29%	64%	89%	94%
**70%**	12%	36%	73%	93%	97%

### 3.2. Discussion

*M. avium* subsp. *avium* and *M. avium* subsp. *hominissuis* are relevant food safety risks in pigs [6,7,8]. With the present MA-ELISA detection of MA risk herds can be done much easier than by the classical incision of lymph nodes in the traditional meat inspection. Infections of the lymph nodes are detected by inspection of the submaxillary lymph nodes after incision within the traditional meat inspection in pigs. A recent review showed that during meat inspection in Germany malformations were detected in only 0.22% of slaughtered pig carcasses (Federal Statistical Office of Germany, 2007; according to BfR report). Caseous malformations in porcine lymph nodes and sometimes in kidneys, liver and spleen can be caused by mycobacteria [9,18,19], but most of them originate from *Rhodococcus equi* infections [12,20,21]. On the other hand, other studies showed that lymph nodes without any lesions can harbour MA [20,22]. Henceforth, the incision of submaxillary lymph nodes in the traditional meat inspection appears to be a non-sensitive and a non-specific test.

In the present study a serological screening for MA infections as alternative method was tested to identify MA positive herds at slaughter. The number of MA positive carcasses was 1.01% and 1.73% in respectively the Dutch and German pig population. These proportions of positive carcasses are comparable to the prevalence of granulomatous malformations in lymph nodes seen at pathological examination, 1.85% by Fischer [23], 0.89% by Meyer *et al.* [24] and 0.48% by Lücker *et al.* [25] of which about one third showed to be bacteriological positive for MA.

The results of the present study show, that serological screening for MA infections has the capacity to identify bacteriological MA positive herds. Screening results showed that 0.5% of the Dutch herds and 17.4% of the German herds had two or more positive samples out of 36 analysed blood samples. These figures also show that MA infection in pigs occurs at a low level. Additionally they show that the prevalence of MA infections differs across populations. There was a higher level of positive samples and herds in the German population compared to the Dutch. As recent studies show [10,11], peat, that is usually used as a feed supplement may be contaminated with MA. In German breeder herds peat is more frequently supplied than in Dutch ones (data not shown) which possibly explains the differences.

The applied MA-ELISA was validated on tuberculation-confirmed MA positive farms. The validation results showed, that the sensitivity of an individual test was low, *i.e*., varying sensitivities were found with an average of 4.3%. Nevertheless, it was shown that approximately 20% of bacteriologically positive herds can be identified when 36 blood samples are tested and at least two samples need to be positive above PP 20 in the ELISA. An improvement of the MA-ELISA test sensitivity seems achievable, as in experimentally infected pigs [13] and in some of the field farms these higher sensitivities were observed. The low average sensitivity might be due to presence of infections with other MA serotypes [26] that have insufficient cross-immunity toward the antigens used in the test. Additional antigens could be added to the MA-ELISA test to improve its performance.

Besides the fact that screening in pig blood collected at slaughter can efficiently be done, there are important advantages of omitting incision of the lymph nodes within meat inspection. Firstly, cross contamination of salmonella due to incision is prevented [15,27]. Secondly, in the supply chain meat inspection system, where this serology is used to categorise herds, much more effort is done to control MA with increased biosecurity standards and follow-up at high risk farms [17]. This prevention of infection with MA in swine at farm level has not been an active constituent in the traditional meat inspection, which is an end of line check only.

## 4. Conclusions

It can be concluded that a population-wide screening for the presence of MA antibodies is capable of identifying pig populations that are at higher risk for MA infection. The validation results of the applied ELISA indicate that positive farms will not in all cases be identified at first instance. However, on the farms that are identified, MA is actively prevented from entering the food chain. Positive MA test results can be reported back to the pig producers and, additionally, control measures and corrective actions can take place at farm level. In this way the overall prevalence in the supplying herds will be reduced. Moreover, abolishment of incision of the lymph nodes prevents cross contamination with salmonella, improving food safety level.
